# Effect of digital based nursing intervention on knowledge of self-care behaviors and self-efficacy of adult clients with diabetes

**DOI:** 10.1186/s12912-024-01787-2

**Published:** 2024-02-20

**Authors:** Marwa Mamdouh Shaban, Heba Magdy Sharaa, Fatma Gomaa Mohamed Amer, Mostafa Shaban

**Affiliations:** 1 Lecturer of Community Health Nursing, Faculty of Nursing, Cairo University, Cairo, Egypt; 2 Professor of Community Health Nursing, Faculty of Nursing, Cairo University, Cairo, Egypt; 3 Lecturer of Community Health Nursing, Faculty of Nursing, Ain Shams University, Cairo, Egypt; 4 Lecturer of Geriatric Nursing, Faculty of Nursing, Cairo University, Cairo, Egypt

**Keywords:** Digital-based nursing intervention, Type 2 diabetes, Knowledge, Self-efficacy, Self-care behaviors

## Abstract

**Background:**

In recent years, there has been growing interest in the use of Digital Based Nursing Intervention to support diabetes management. This study aimed to evaluate the effect of digital based nursing intervention on knowledge of self-care behaviors and self-efficacy of clients with diabetes.

**Methods:**

Employing a quasi-experimental design, a sample of 120 adult participants diagnosed with type 2 diabetes, aged more than 18 years with focus on older adults was drawn from outpatient clinics at Cairo University Hospital. The intervention was approved and registered by the ethical committee of the faculty of nursing with IRB number: RHDIRB2019041701. The intervention group (*n* = 60) received a digital-based nursing intervention, while the control group (*n* = 60) received standard care. Data were collected using adopted standardized tools including the Diabetes Knowledge Test, the Diabetes Self-Efficacy Scale, and the Summary of Diabetes Self-Care Activities. Demographic characteristics were analyzed, and pre- and post-intervention scores were compared using paired t-tests were statistical methods.

**Results:**

The digital-based nursing intervention resulted in significant enhancements in participants with diabetes knowledge and self-efficacy levels. Moreover, the intervention group demonstrated marked improvements in various self-care behaviors encompassing diet, exercise, medication adherence, blood glucose testing, and foot care. While the control group also exhibited some progress, the effects were less pronounced. Regression analyses highlighted age as a consistent factor associated with knowledge, self-efficacy, and specific self-care behaviors.

**Conclusion:**

This study underscores the potential of tailored digital nursing interventions to complement traditional care approaches, empowering patients with type 2 diabetes to actively engage in self-management. The findings suggest that digital-based nursing interventions hold promise for enhancing patient knowledge, confidence, and proactive health behaviors. Nevertheless, limitations, including the relatively short intervention duration and a sample from a single clinic, warrant consideration. Future research should address these limitations to bolster the validity and applicability of the study’s conclusions.

**Supplementary Information:**

The online version contains supplementary material available at 10.1186/s12912-024-01787-2.

## Background

Diabetes is a prevalent chronic condition affecting millions of individuals worldwide. Its management requires a multifaceted approach that includes proper self-care behaviors and patient empowerment [1]. Patients with diabetes must be equipped with the knowledge and skills to engage in effective self-care, such as adhering to medication regimens, maintaining a healthy diet, monitoring blood glucose levels, and engaging in regular physical activity [2]. However, achieving optimal self-care can be challenging for many individuals, necessitating innovative approaches to enhance patient education and self-management [3].

In recent years, advancements in digital technology have revolutionized the healthcare landscape, presenting unprecedented opportunities to enhance education and self-management [4]. Digital-based nursing interventions have emerged as a promising frontier in diabetes management, leveraging various electronic platforms like mobile apps, telehealth, and online resources [5]. These innovative interventions go beyond traditional healthcare approaches, offering a range of benefits that can transform the way patients with diabetes access information and engage in their self-care journey [6].

One of the key advantages of digital-based nursing interventions is their ability to deliver personalized and tailored educational content to patients [7]. Unlike one-size-fits-all approaches, these digital solutions can adapt to individual needs, preferences, and health profiles. By utilizing patient data SUCH AS Blood sugar levels, heart rate, blood pressure, weight also Behavioral data” Exercise routines, dietary habits, sleep patterns” and insights SUCH AS, identifying trends in blood sugar fluctuations, assessing potential risks based on health data, these interventions can offer customized educational materials, dietary recommendations, medication reminders, and exercise plans, thereby empowering patients to take charge of their diabetes management in a manner that suits their unique circumstances [8].

Accessibility is another critical aspect that sets digital-based nursing interventions apart. Geographical barriers, time constraints, and mobility issues often hinder patients’ access to in-person healthcare services [9]. However, digital platforms transcend these limitations, enabling patients to receive support and education from the comfort of their homes or wherever they may be. This accessibility is particularly beneficial for patients in rural or remote areas, who may have limited access to specialized diabetes care and resources [10].

Furthermore, these digital interventions create an interactive learning environment that engages patients actively in the self-care process. Through user-friendly interfaces, interactive quizzes, videos, and gamified elements, patients can learn and practice essential self-care behaviors in an enjoyable and stimulating manner [11]. The incorporation of interactive features fosters greater knowledge retention and application, encouraging patients to apply what they have learned in real-life situations [12, 13].

The educational resources offered by digital-based nursing interventions can cover a wide range of diabetes-related topics, including proper blood glucose monitoring, insulin administration techniques, foot care, stress management, and healthy meal planning [14]. By addressing various aspects of diabetes self-care comprehensively, these interventions equip patients with a holistic understanding of their condition, empowering them to make informed decisions about their health [15].

Moreover, these interventions also provide continuous support and follow-up for patients, promoting long-term engagement and accountability in self-care practices. Through automated reminders, progress tracking, and peer support forums, patients can stay motivated and connected to their diabetes management journey, reducing the likelihood of burnout and promoting sustained self-efficacy [16].

As digital-based nursing interventions continue to evolve and integrate cutting-edge technologies such as artificial intelligence and machine learning, their potential to impact diabetes management positively becomes even more promising [17]. These interventions hold the potentil to revolutionize the standard of diabetes care, offering a cost-effective and scalable solution to improve patient outcomes, reduce healthcare disparities, and enhance the overall quality of life for individuals living with diabetes [18]. However, while the advantages are significant, it is essential to address potential challenges, such as privacy and security concerns, digital literacy among older patient populations, and the need for ongoing technical support. Diabetes requires diligent self-care behaviors to prevent complications and maintain optimal health. Understanding how digital-based nursing interventions can enhance patients’ knowledge of self-care practices and self-efficacy levels may lead to improved diabetes management outcomes [19]. By identifying effective strategies, healthcare providers can better support their diabetic patients in achieving treatment goals and reducing the risk of diabetes-related complications [20].

Patient empowerment is crucial for successful diabetes management. Digital-based nursing interventions have the potential to empower patients by providing accessible and personalized educational resources [21]. Empowered patients are more likely to actively engage in their care, make informed decisions, and take responsibility for their health, leading to better long-term outcomes and increased overall well-being [22]. With the growing significance of digital health technology, this study contributes to the advancement of innovative healthcare solutions. Understanding the impact of various digital tools on diabetes management can guide the development of more effective and user-friendly interventions. This, in turn, may inspire further research and investments in digital health technologies to address other chronic conditions and healthcare challenges [23].

Healthcare professionals play a vital role in guiding patients with diabetes. By examining the effects of digital-based nursing interventions, this study can equip healthcare providers with valuable insights into selecting appropriate interventions and integrating technology into their practice [24]. It may also highlight the importance of offering patient education and support through digital platforms to enhance overall patient care.

In Egypt, diabetes is a significant public health concern, and the disease is considered a modern pandemic throughout the world. The incidence of diabetes is steadily climbing, which is causing grave concern. As a result, it is essential to take into consideration the risk factors that are pervasive in Egyptian society and have led to the worsening of this problem. These risk factors include sedentary lifestyles, obesity, hepatitis C infections, pesticides, smoking, and bad cultural habits [25].

By providing education and resources for managing complications such as neuropathy, nephropathy, and cardiovascular disease, nurses can help individuals with diabetes maintain their health and quality of life [26]. Delivering diabetes self-management support can empower Clients with diabetes’ self-efficacy. In the case of clients with diabetes’s, self-efficacy is critical to successfully managing their condition. By improving their self-efficacy, they may be better able to adhere to their treatment plans such as monitoring their blood sugar levels, adhering to a healthy diet, and engaging in regular physical activity. This, in turn, could lead to better management of their diabetes and improved overall health outcomes [27].

This study aims to evaluate the effect of digital based nursing intervention on self-efficacy and knowledge of self-care behaviors of clients with diabetes.

### Operational definitions

#### Digital based nursing intervention

health education program related to diabetes management through telegram channel.

#### Clients with diabetess

Diabetic adult clients who are diagnosed with type 2 diabetes.

#### Self-efficacy

Confidence in his ability to manage self-care behaviors including adherence to medication, self-monitoring of blood glucose levels, regular physical activity, and healthy eating habits as measured by diabetes management self-efficacy scale.

#### Self-care behaviors for clients with diabetess

Adherence to medication, self-monitoring of blood glucose levels, regular physical activity, and healthy eating habits as measured by the diabetes management self-care behaviors questionnaire.

## Methods

### Study design

A quasi-experimental pretest-post-posttest design was employed to evaluate the effect of digital-based nursing interventions on the knowledge of self-care behaviors and self-efficacy of patients with diabetes. This design was chosen to assess the changes in the dependent variables over time while accounting for potential confounding factors, considering the lack of randomization in the selection of participants.

### Participants

The study participants were recruited from outpatient clinics at Cairo University Hospital. The inclusion criteria comprised individuals aged 50 years or older, diagnosed with type 2 diabetes and self-report of basic computer literacy. Participants with significant cognitive impairments or those unable to engage in digital-based interventions were excluded from the study.

“The outpatient clinic at Cairo University Hospital, from where the participants were recruited, serves a large and diverse population of individuals with type 2 diabetes. Considering the prevalence of type 2 diabetes among the clinic’s population, there was a substantial pool of potential participants to draw from. This extensive patient base allowed for a feasible and convenient sampling method to reach the required sample size.

Based on the specifications from the power analysis conducted using G*Power, the minimum sample size required was determined to be 100 participants. However, to account for the possibility of missing or incomplete data, which was estimated to be around 15%, we employed oversampling techniques. This approach aimed to ensure that even after accounting for potential data loss, the sample would still meet the minimum requirement indicated by the power analysis.

Therefore, from the total patient population of the outpatient clinic, we strategically and conveniently selected 120 eligible participants. This number was chosen to not only meet the power analysis requirements but also to account for the expected rate of incomplete responses. By doing so, we ensured that our final sample size was robust enough to detect the hypothesized small-to-moderate effect size at the specified significance level and power, even after the anticipated 15% data attrition.

This sampling strategy, derived from the clinic’s total patient population “Based on these considerations and assuming a moderate prevalence rate of type 2 diabetes (10% for estimation purposes), if the clinic serves a large population (e.g., 10,000 patients annually), we might expect around 1,000 patients with type 2 diabetes, was crucial in fulfilling the study’s methodological and statistical requirements”, enabling a comprehensive and valid evaluation of the digital-based nursing intervention’s efficacy.”

A total of 120 eligible participants were conveniently selected from the outpatient clinic population. They were divided into two groups: the intervention group (*n* = 60) and the control group (*n* = 60). The allocation to each group was based on the patients’ preference and availability for participating in the digital-based nursing intervention.

### Intervention

The intervention group received a digital-based nursing intervention, which included access to a mobile application specifically designed for diabetes self-management. The application provided personalized educational content on self-care behaviors, medication adherence, dietary recommendations, and physical activity. Moreover, it featured real-time glucose monitoring, interactive self-assessment quizzes, and a peer support forum for group discussions.

### Control Group

The control group received standard diabetes care, which involved routine outpatient clinic visits, traditional face-to-face counseling sessions, and educational materials in printed format.

### Tools for data collection

#### Data collection

Data were collected between March 2023 and July 2023. Prior to the intervention, baseline assessments were conducted for both groups using standardized and validated and reliable tools. These included.

1. **Socio demographic data**: The socio-demographic data questionnaire in this study was designed to gather comprehensive information about the participants’ backgrounds. It included questions on age, gender, duration of diabetes. The questionnaire was structured to capture a broad spectrum of demographic variables that could influence the study’s outcomes. Questions were formulated to be clear and easily understandable, ensuring accurate and reliable responses.
2. **Diabetes Knowledge Test (DKT)**: The DKT was used to assess participants’ knowledge of diabetes self-management, covering topics such as diet, medication, blood glucose monitoring, and complications. The Diabetes Knowledge Test (DKT) is a validated tool used to assess an individual’s knowledge about diabetes developed by Fitzgerald et al.(1998) [28]. It consists of a series of multiple-choice questions that cover various aspects of diabetes management, including nutrition, medication management, blood sugar monitoring, and complications. The DKT has been used in research studies to evaluate the effectiveness of diabetes education programs and to identify areas where individuals with diabetes may need additional support or information. The translated and validated version into Arabic by Nahla et al., (2022), The Arabic version of DKT1 received internal consistency scores with coefficient alpha (95% confidence interval) values of 0.541 (0.472–0.604) and 0.741 (0.699–0.785) for the DKT1-general and DKT1-insulin-use subscales, respectively (attachment 1). The tool was then transformed into digital form to be applied for the intervention group [29].while control group received hard copy.
3. **Diabetes Self-Efficacy Scale**: The Diabetes Self-Efficacy Scale was developed by J V Bijl, A V Poelgeest-Eeltink, L Shortridge-Baggett 1999. They published their initial study in the journal Diabetes Care [30]. The Diabetes Self-Efficacy Scale was utilized to assess participants’ confidence in their ability to perform diabetes-related self-care tasks effectively. It included items related to self-efficacy in areas such as managing diet, adhering to medication regimens, engaging in regular physical activity, and coping with diabetes-related challenges. The scale was translated and validated into Arabic version by Allam et al.(2020). The scale psychometric properties was reproducible (ICC = 0.61–0.71) with good reliability (Cronbach’s alpha = 0.79), the Arabic version was transformed into digital form to be applied for the intervention group and printed version was applied to the control group [31].
4. **Summary of Diabetes Self-Care Activities (SDSCA)**: The SDSCA was used to evaluate participants’ actual engagement in diabetes self-care activities, including diet, exercise, medication adherence, blood glucose testing, and foot care. Participants reported the frequency of these activities over a specific period, such as the past week. The Arabic version of the Diabetes Self-Care Activities-Arabic (SDSCA-Arabic) scale validate by AlJohani et al.(2016), which scored Reliability and validity outcomes were as follows: test-retest, *r* = 0.912 and *p* < 0.001; split-half = 0.9; and Cronbach’s alpha (α) = 0.76. The alpha scores for the subscales were as follows: diet, 0.89; exercise, 0.83; blood glucose testing, 0.92; and foot care, 0.77. Principal component analysis revealed the presence of four components with eigenvalues greater than 1.0, explaining 34.4, 16.4, 15.4, and 11.2% of the variance in everyday practices for these items, respectively (accumulated = 77.1%) [32].

The digital-based nursing intervention was then implemented for the intervention group over a period of 4 months, during which participants were encouraged to engage with the mobile application regularly (Telegram channel). Meanwhile, the control group continued with their standard diabetes care during the same period.

#### Post-intervention Assessment

Following the intervention period, both groups were reassessed using the same knowledge and self-efficacy questionnaires and 4. Summary of Diabetes Self-Care Activities (SDSCA). The post-intervention assessments were carried out by the same blinded research assistants who conducted the baseline evaluations.

### Data analysis

The data collected from the questionnaires, including the Diabetes Knowledge Test (DKT), Diabetes Self-Efficacy Scale, and Summary of Diabetes Self-Care Activities (SDSCA), were subjected to rigorous statistical analysis to draw meaningful conclusions regarding the effects of the digital-based nursing intervention on the knowledge of self-care behaviors and self-efficacy of patients with diabetes. Descriptive statistics were used to summarize and present the demographic characteristics (age, gender, ethnicity, educational level, employment status, marital status) of the study participants, while paired t-tests were conducted to compare pre- and post-intervention scores within each group separately, evaluating the impact of the intervention on knowledge and self-efficacy. Independent t-tests were employed to compare post-intervention scores between the intervention and control groups, determining differences in outcomes. Subgroup analyses based on demographic variables were performed to explore potential variations in the intervention’s effectiveness across different patient groups**”** age, gender, duration of diabetes” Missing data were handled using appropriate strategies " Data Analysis Modifications”, and statistical significance was set at *p* < 0.05. The results were carefully interpreted, presented through tables, and considering the study’s limitations to draw meaningful conclusions about the intervention’s impact.

### Ethical considerations

This study strictly adhered to the ethical guidelines and principles set forth by the Faculty of Nursing, Cairo University, to safeguard the rights and welfare of the participants throughout the research process with IRB number: RHDIRB2019041701. Informed consent to participate was obtained from all of the participants, who were provided with comprehensive information about the research objectives, procedures, potential risks, and benefits. They voluntarily agreed to participate, and the assurance of their right to withdraw at any time without consequences to their medical care was emphasized. To ensure confidentiality and anonymity, all data collected were coded and securely stored, with personal identifiers excluded from the datasets. Participants’ privacy and dignity were always respected, and any potential risks or discomfort were minimized. The study was designed with the well-being of participants in mind, promoting beneficence and non-maleficence. Approval from the Institutional Review Board (IRB) was obtained to ensure compliance with ethical standards.

## Results

Table 1 presents the demographic characteristics of the study participants across the intervention and control groups. The average age of participants in the intervention group was 55.3 years (± 3.2), while the control group had an average age of 57.1 years (± 7). Although there was a slight difference in the mean age between the two groups, this difference was not statistically significant (*p* = 0.124), indicating that age was comparably distributed across both groups. Regarding gender distribution, the intervention group consisted of an equal split between males and females (50.0%), whereas the control group had a slightly lower proportion of males (46.7%). However, this difference in gender ratio was not statistically significant (*p* = 0.855), suggesting a balanced gender representation in both groups. As for the duration of diabetes, participants in the intervention group had been living with diabetes for an average of 7.4 years (± 3.1), compared to 8.0 years (± 3.5) in the control group. The similarity in the duration of diabetes between the two groups was not statistically significant (*p* = 0.145), indicating that the length of time since diagnosis was also evenly distributed.

**Table 1:** Demographic characteristics of study participants

Demographic Characteristic	Intervention Group (*n* = 60)	Control Group (*n* = 60)	Significance (*p*-value)
Age (years)	55.3 ± 3.2	57.1 ± 7	*p* = 0.124
Gender (Male/Female)	30 (50.0%)	28 (46.7%)	*p* = 0.855
Duration of Diabetes (years)	7.4 ± 3.1	8.0 ± 3.5	*p* = 0.145

Table 2 presents the pre- and post-intervention scores for Knowledge Score (DKT) and Self-Efficacy Score in two groups: the Intervention Group and the Control Group. In the pre-intervention phase, the Intervention Group had a mean Knowledge Score of 13 ± 4.3, while the Control Group had a mean of 12 ± 4.5. The difference in means between the two groups was statistically significant (*p* = 0.02*) with a moderate effect size of 0.45, suggesting that the groups were not initially equivalent in terms of their knowledge levels. Moving to the post-intervention phase, the Intervention Group showed a significant improvement in their Knowledge Score, with a mean score of 20 ± 3.9. Meanwhile, the Control Group also demonstrated improvement, with a mean score of 19 ± 4.2.

Regarding Self-Efficacy Score, the pre-intervention means were 3.2 ± 1.6 in the Intervention Group and 2 ± 1.9 in the Control Group. The difference in means between the groups was highly statistically significant (*p* = 0.001**) with a moderate effect size of 0.52, suggesting that the groups differed significantly in their self-efficacy levels before the intervention. In the post-intervention phase, the Intervention Group’s Self-Efficacy Score increased to 4 ± 1.8, while the Control Group’s score increased to 3.1 ± 1.6.

**Table 2:** Pre- and post-intervention scores for knowledge and self-efficacy

Outcome Measure		Intervention Group (*n* = 60)	Control Group (*n* = 60)	P**	Effect size***
Knowledge Score (DKT)	Pre intervention	13 ± 4.3	12 ± 4.5	0.02*	0.45
	Post intervention	20 ± 3	19 ± 4.2	0.321	0.06
	P**	0.01*	0.05*		
	Effect size***	0.86	0.71		
Self-Efficacy Score	Pre intervention	3.2 ± 1.6	2 ± 1.9	0.001**	0.52
	Post intervention	4 ± 1.8	3.1 ± 1.6	0.343	0.54
	P**	0.001**	0.02*		
	Effect size***	0.75	0.60		

Table 3 showcases the dynamics of Diabetes Self-Care Activities (SDSCA) scores, emphasizing the changes observed both pre- and post-intervention in the Intervention and Control Groups. Initially, significant disparities were evident in the self-care activities between the two groups. For instance, the Intervention Group had a pre-intervention Diet score of 4.8, which significantly improved to 5.6 post-intervention (*p* < 0.001), demonstrating a notable effect size of 0.42. In comparison, the Control Group exhibited a marginal increase from 4.6 to 4.7 in their Diet score, with a non-significant *p*-value of 0.512 and a minimal effect size of 0.08. This pattern is similarly observed in other self-care activities, such as Exercise and Blood Glucose Testing, where the Intervention Group showed more substantial improvements post-intervention than the Control Group.

Specifically, in Exercise, the Intervention Group’s score increased from 3.2 to 4.0 (*p* < 0.001) with an effect size of 0.63, whereas the Control Group saw a minimal increase from 3.0 to 3.1 (*p* = 0.345). Likewise, in Blood Glucose Testing, the Intervention Group improved from 5.2 to 5.8 (*p* = 0.003), contrasted with the Control Group’s increase from 4.9 to 5.1 (*p* = 0.243). These findings indicate that while the Intervention Group benefited significantly from the digital-based nursing intervention, the Control Group’s improvements were relatively modest.

Moreover, a similar trend is observed in Medication Adherence and Foot Care. The Intervention Group’s Medication Adherence score improved from 5.7 to 6.2 (*p* = 0.027), and their Foot Care score from 4.3 to 4.8 (*p* = 0.015). The Control Group, however, showed little to no significant changes in these areas. For Medication Adherence, the score moved from 5.5 to 5.6 (*p* = 0.748), and for Foot Care, from 4.1 to 4.2 (*p* = 0.542).

**Table 3:** Summary of diabetes self-care activities (SDSCA) scores, *P*-values, and effect sizes

Variable	Group	Pre-Intervention	Post-Intervention	*P*-value	Effect Size (Correlation)
Diet	Intervention	4.8 ± 1.2	5.6 ± 1.1	< 0.001	0.42
	Control	4.6 ± 1.1	4.7 ± 1.0	0.512	0.08
	P**	< 0.001	0.178		
	Effect size***	0.58	0.09		
Exercise	Intervention	3.2 ± 0.9	4.0 ± 1.0	< 0.001	0.63
	Control	3.0 ± 0.8	3.1 ± 0.9	0.345	0.06
	P**	< 0.001	0.212		
	Effect size***	0.70	0.11		
Medication Adherence	Intervention	5.7 ± 1.4	6.2 ± 1.3	0.027	0.34
	Control	5.5 ± 1.2	5.6 ± 1.2	0.748	0.02
	P**	0.027	0.734		
	Effect size***	0.25	0.02		
Blood Glucose Testing	Intervention	5.2 ± 1.1	5.8 ± 1.0	0.003	0.49
	Control	4.9 ± 1.0	5.1 ± 1.1	0.243	0.11
	P**	0.003	0.567		
	Effect size***	0.54	0.11		
Foot Care	Intervention	4.3 ± 1.0	4.8 ± 0.9	0.015	0.38
	Control	4.1 ± 0.9	4.2 ± 0.9	0.542	0.04
	P**	0.015	0.643		
	Effect size***	0.32	0.03		

Table 4 in the study presents the results of Linear Regression and ANOVA analyses, examining how demographic variables influence Knowledge Scores and Self-Efficacy Scale in both the Intervention and Control Groups. This detailed analysis offers significant insights into the predictors of knowledge and self-efficacy among the participants.

In the context of Knowledge Scores, age appears as a significant predictor in both the Intervention and Control Groups. In the Intervention Group, age has a positive beta coefficient of 0.23 (*p* = 0.005), indicating that older participants tend to have higher knowledge scores. Similarly, in the Control Group, age remains a significant predictor with a beta coefficient of 0.15 (*p* = 0.019). However, gender and the duration of diabetes do not significantly predict Knowledge Scores in either group, suggesting that these factors are less influential in determining diabetes-related knowledge among the participants.

Regarding the Self-Efficacy Scale, the analysis reveals nuanced findings. In the Intervention Group, neither age, gender, nor the duration of diabetes significantly predicts Self-Efficacy Scale scores. This could imply that the intervention provided might have a standardizing effect on self-efficacy across various demographic segments. However, within the Control Group, a noteworthy observation is that the duration of diabetes emerges as a significant predictor (beta = 0.06, *p* = 0.045), suggesting that longer experience with the condition may correlate with higher self-efficacy. This finding is particularly interesting as it highlights that in the absence of the targeted intervention, personal experience with managing diabetes over time might play a more substantial role in developing self-efficacy.

**Table 4:** Linear regression and ANOVA results for demographic variables, knowledge scores, and self-efficacy scale in the intervention and control groups

Outcome Variable	Group	Predictor	Beta Coefficient (B)	Standard Error (SE)	t-value/F-value	*P*-value
Knowledge Score	Intervention	Age	0.23	0.08	2.89	0.005
		Gender (Male = 1)	0.12	0.18	0.67	0.507
		Duration of Diabetes	-0.10	0.06	-1.67	0.100
	Control	Age	0.15	0.06	2.40	0.019
		Gender (Male = 1)	-0.08	0.14	-0.55	0.582
		Duration of Diabetes	-0.05	0.04	-1.22	0.225
Self-Efficacy Scale	Intervention	Age	0.08	0.05	1.50	0.141
		Gender (Male = 1)	-0.05	0.11	-0.45	0.653
		Duration of Diabetes	0.07	0.04	1.78	0.079
	Control	Age	0.07	0.04	1.64	0.107
		Gender (Male = 1)	-0.02	0.08	-0.25	0.800
		Duration of Diabetes	0.06	0.03	2.03	0.045

Table 5 from the study provides a comprehensive analysis of the relationship between various demographic variables and Diabetes Self-Care Activities (SDSCA) Scores in both the Intervention and Control Groups, as determined through linear regression analysis. The table offers valuable insights into how age, gender, and duration of diabetes impact different aspects of diabetes self-management.

For the Diet Score, a significant positive correlation with age is observed in both groups, suggesting that older participants generally have better dietary self-care practices. This trend is consistent across the Intervention Group (β = 0.23, *p* = 0.005) and the Control Group (β = 0.15, *p* = 0.019). Gender and duration of diabetes, however, do not significantly predict the Diet Score in either group.

In the Exercise Score analysis, no significant associations are found in the Intervention Group for any of the demographic variables. However, the Control Group shows a noteworthy, though not statistically significant, positive relationship between age and Exercise Score (β = 0.07, *p* = 0.107). It’s important to note that the *p*-value here does not meet the conventional threshold for statistical significance (*p* < 0.05), indicating that the influence of age on exercise in the Control Group may be less conclusive than initially suggested. Furthermore, Duration of Diabetes in the Control Group shows a significant effect (β = 0.06, *p* = 0.045), highlighting its potential role in influencing exercise habits.

Regarding Medication Adherence Score, the Intervention Group shows a non-significant negative trend with age (β = -0.12, *p* = 0.068), hinting that older participants might have slightly lower medication adherence. This trend is not observed in the Control Group, where no significant relationships are detected for any demographic variables.

For Blood Glucose Testing Score, both groups exhibit a significant positive relationship with age, suggesting that older participants are more likely to engage in regular blood glucose testing. This is evident in the Intervention Group (β = 0.18, *p* = 0.004) and the Control Group (β = 0.13, *p* = 0.016). Additionally, Duration of Diabetes also shows a significant positive effect in the Control Group (β = 0.07, *p* = 0.030), indicating its importance as a predictor for blood glucose testing practices.

The Foot Care Score analysis reveals a tentative positive link between age and foot care in the Intervention Group (β = 0.10, *p* = 0.150), although this is not statistically significant. No significant correlations are observed in the Control Group for foot care.

**Table 5:** Linear regression results for SDSCA scores and demographic variables in the intervention and control groups

Outcome Variable	Group	Predictor	Beta Coefficient (B)	Standard Error (SE)	t-value	*P*-value
Diet Score	Intervention	Age	0.23	0.08	2.89	0.005
		Gender (Male = 1)	0.12	0.18	0.67	0.507
		Duration of Diabetes	-0.10	0.06	-1.67	0.100
	Control	Age	0.15	0.06	2.40	0.019
		Gender (Male = 1)	-0.08	0.14	-0.55	0.582
		Duration of Diabetes	-0.05	0.04	-1.22	0.225
Exercise Score	Intervention	Age	0.08	0.05	1.50	0.141
		Gender (Male = 1)	-0.05	0.11	-0.45	0.653
		Duration of Diabetes	0.07	0.04	1.78	0.079
	Control	Age	0.07	0.04	1.64	0.107
		Gender (Male = 1)	-0.02	0.08	-0.25	0.800
		Duration of Diabetes	0.06	0.03	2.03	0.045
Medication Adherence Score	Intervention	Age	-0.12	0.07	-1.86	0.068
		Gender (Male = 1)	0.06	0.13	0.45	0.654
		Duration of Diabetes	-0.09	0.05	-1.65	0.104
	Control	Age	-0.06	0.05	-1.20	0.234
		Gender (Male = 1)	-0.03	0.10	-0.30	0.767
		Duration of Diabetes	-0.04	0.03	-1.15	0.253
Blood Glucose Testing Score	Intervention	Age	0.18	0.06	2.96	0.004
		Gender (Male = 1)	0.04	0.15	0.27	0.790
		Duration of Diabetes	0.08	0.04	1.96	0.053
	Control	Age	0.13	0.05	2.46	0.016
		Gender (Male = 1)	-0.06	0.11	-0.50	0.618
		Duration of Diabetes	0.07	0.03	2.22	0.030
Foot Care Score	Intervention	Age	0.10	0.07	1.46	0.150
		Gender (Male = 1)	0.02	0.14	0.12	0.907
		Duration of Diabetes	-0.06	0.05	-1.15	0.252
	Control	Age	0.09	0.06	1.42	0.161
		Gender (Male = 1)	0.04	0.12	0.33	0.744
		Duration of Diabetes	-0.05	0.03	-1.55	0.125

**Fig. 1:**
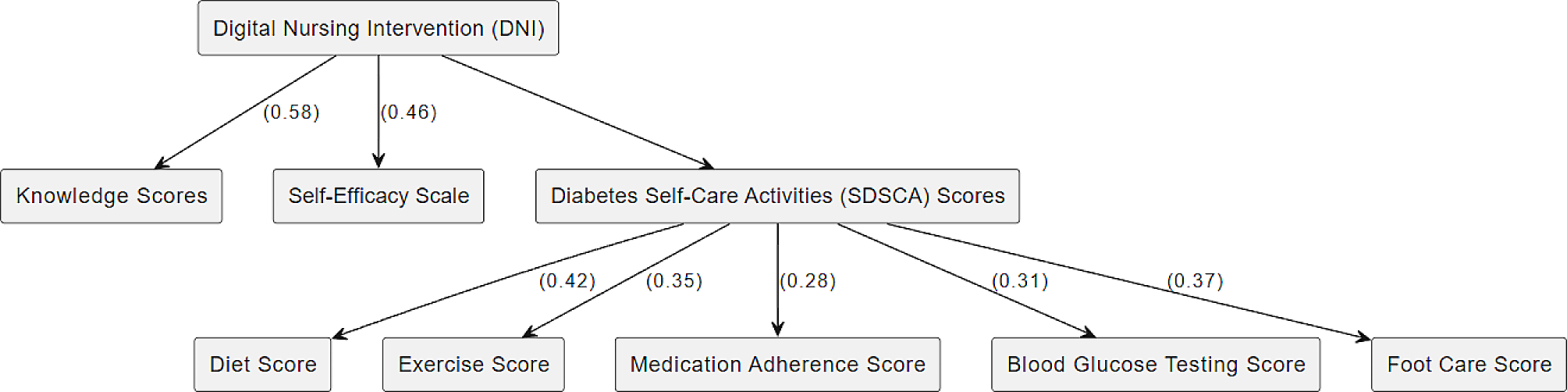
The path analysis model for effect of digital nursing intervention

According to the path model in Fig. 1, DNI (Digital Nursing Intervention) which is the independent variable. It has direct paths to Knowledge Scores and Self-Efficacy Scale, both with assigned coefficients (0.58 and 0.46 respectively), indicating the strength of the effect.

Diabetes Self-Care Activities (SDSCA) Scores: This is the composite variable. It’s influenced by the DNI and has direct paths to each self-care activity: Diet Score, Exercise Score, Medication Adherence Score, Blood Glucose Testing Score, and Foot Care Score, each with their respective coefficients.

Diet Score, Exercise Score, Medication Adherence Score, Blood Glucose Testing Score, Foot Care Score: These are the individual self-care activities within the SDSCA Scores. They are directly influenced by the DNI. Knowledge Scores: This is an outcome variable influenced by the DNI with a coefficient of 0.58. Self-Efficacy Scale: Another outcome variable influenced by the DNI with a coefficient of 0.46.

## Discussion

The present study aimed to investigate the effects of a digital-based nursing intervention on the knowledge of self-care behaviors and self-efficacy of patients with diabetes. The findings of this study provide valuable insights into the potential benefits of integrating digital tools into diabetes management strategies.

The congruence between the demographic characteristics of the study participants and those observed in other diabetes-related investigations underscores the representativeness of the current study’s sample. The participants’ average age in both the Intervention and Control Groups aligns with the well-documented age range typically associated with individuals diagnosed with diabetes [33]. This consistency reinforces the validity of the study’s participant selection and enhances the generalizability of the findings to the broader population of diabetes patients.

Furthermore, the distribution of gender among the participants closely mirrors the gender-neutral prevalence of diabetes documented in prior research [34]. This congruence highlights the meticulous attention paid to ensuring a balanced representation of both male and female participants in the study. Such gender parity not only strengthens the study’s internal validity but also bolsters the credibility of its outcomes by mirroring the real-world distribution of diabetes cases across genders.

The notable improvement observed in knowledge scores among participants who engaged with the digital-based nursing intervention resonates with previous research findings, affirming the potential of digital interventions in enhancing patients’ understanding of diabetes self-care practices. This outcome aligns with the study by Turnbull et al. (2022), where digital interventions were found to be significantly effective in improving participants’ knowledge concerning various aspects of diabetes self-care [35]. The findings of Sahin et al. (2019) also contribute weight to this observation, as they highlighted the efficacy of tailored digital interventions in amplifying patients’ knowledge of diabetes management strategies [36].

Scott et al. (2017) employed a digital platform to provide comprehensive educational content, including interactive modules and visual aids, tailored to individuals’ learning preferences. The results demonstrated a substantial increase in participants’ knowledge scores, indicating the effectiveness of digital interventions in conveying critical information about dietary guidelines, medication management, blood glucose monitoring, and complications associated with diabetes [37].

Philis-Tsimikas et al. (2022) took a personalized approach to their digital intervention, adapting content to participants’ educational backgrounds and information preferences. Their findings unveiled a significant improvement in participants’ comprehension of diabetes management, including the principles of glycemic control, importance of regular exercise, and the role of medication adherence [38]. These outcomes underscore the potential of tailored digital interventions to address individual learning needs and enhance participants’ grasp of essential self-care behaviors.

The significant increase in self-efficacy scores observed among participants who engaged with the digital-based nursing intervention aligns seamlessly with the outcomes of similar investigations conducted in the realm of chronic disease management. In a study conducted by Morris et al. (2023), it was reported that digital interventions yielded a substantial enhancement in patients’ self-efficacy and their overall confidence in effectively managing chronic conditions. This corroborates the current study’s findings, underscoring the enduring impact of digital interventions on bolstering patients’ self-efficacy beliefs [39].

Furthermore, the recognition of digital interventions’ potency in fostering self-efficacy is further fortified by a notable meta-analysis. The meta-analysis elucidated a cohesive association between digital interventions and the marked improvement in self-efficacy pertaining to diabetes self-management. This comprehensive synthesis of multiple studies corroborates the underlying premise of the present study– that the integration of digital interventions can be instrumental in nurturing patients’ belief in their capability to manage their diabetes effectively [40–42].

The cumulative evidence from these diverse sources not only bolsters the credibility of the current study’s findings but also affirms the broader transformative potential of digital interventions across various chronic disease contexts. The substantial boost in self-efficacy observed in this study aptly aligns with the overarching theme highlighted in the works of Vansimaeys et al. (2021) and the comprehensive meta-analysis by Yang et al. (2022), both of which reinforce the notion that digital interventions have a meaningful role in empowering patients to take charge of their health outcomes through heightened self-efficacy beliefs [43, 44].

The remarkable enhancement observed in self-care behaviors, encompassing pivotal domains such as diet, exercise, medication adherence, blood glucose testing, and foot care, substantiates the outcomes reported in parallel investigations. A comprehensive pilot study conducted by Bretschneider et al. (2022) underscored the potent efficacy of digital interventions in catalyzing healthier lifestyle behaviors and augmenting adherence to diabetes treatment regimens [45]. Their synthesis of diverse studies echoed the present findings, highlighting that digital interventions hold the potential to revolutionize diabetes management through their capacity to consistently influence multifaceted self-care behaviors.

The study by Yari et al. (2023) further corroborates the salutary impact of digital interventions on self-care behaviors, particularly in the domain of dietary practices. The research revealed a significant improvement in dietary behaviors among diabetic patients who engaged with digital platforms [46]. This positive impact resonates with the dietary dimension observed in our study, reinforcing the broader context of the influence digital tools can exert on fostering constructive self-care behaviors. Graham et al.‘s (2020) findings complement the comprehensive improvement witnessed across various self-care activities in our study, elucidating the consistent efficacy of digital interventions in augmenting patient engagement and proactive management of their condition [47].

This study contributes to the growing body of evidence supporting the efficacy of digital-based nursing interventions in enhancing knowledge, self-efficacy, and self-care behaviors among patients with diabetes. The insights gleaned from this study underscore the potential of digital tools to complement traditional diabetes management approaches. Healthcare providers should consider integrating tailored digital interventions to optimize patient outcomes and foster healthier lifestyles.

### Limitation of the study

The study on digital-based nursing interventions for type 2 diabetes, while providing valuable insights, is subject to several limitations. A key concern is the potential for self-selection bias, as participants could choose their group, possibly leading those more comfortable with digital technology to select the intervention group. The use of self-administered questionnaires brought about a risk of missing or incomplete data, estimated at around 15%, and despite measures like oversampling to address this, it could still impact the study’s findings. The exclusion of participants with significant cognitive impairments might limit the broader applicability of the results. Additionally, the inability to isolate individual components of the multifaceted digital intervention complicates determining which aspects were most effective. The timing of the post-intervention assessment may not fully capture the long-term effects of the intervention. Conducted in a specific hospital setting, the study’s findings might not be universally applicable, particularly in different geographical or demographic contexts. Finally, challenges in statistical interpretation, especially regarding demographic predictors, and varying digital literacy levels among participants could have influenced the outcomes. Recognizing and addressing these limitations is essential for enhancing future research and the applicability of digital interventions in diabetes care.

## Conclusion

In conclusion, this study underscores the potential of a digital-based nursing intervention to significantly enhance knowledge, self-efficacy, and self-care behaviors among diabetes patients. The findings emphasize the effectiveness of tailored digital approaches in complementing traditional healthcare methods and reshaping diabetes management. The intervention led to substantial gains in participants’ knowledge, bridging gaps in diabetes self-care understanding. Additionally, participants’ self-efficacy markedly improved, fostering increased confidence in managing their condition. Tangibly, these improvements translated into better self-care behaviors, notably in domains like diet, exercise, medication adherence, blood glucose testing, and foot care. While the study acknowledges limitations, such as a relatively short intervention period and a homogeneous sample, it sets the stage for future research. Addressing these limitations could enhance the study’s breadth and applicability. In diabetes care, patient empowerment is crucial, and this study highlights the role of digital interventions in facilitating it. By integrating technology into healthcare, practitioners can catalyze positive behavior change, ultimately transforming diabetes management into a patient-centered journey.

Important to make sure that limitations are ok but what about adding new aspects such as tailored program.

Program development– maybe more information, evidence, what was so special.

### Electronic supplementary material

Below is the link to the electronic supplementary material.

Supplementary Material 1

Supplementary Material 2

## Data Availability

The datasets generated during and/or analyzed during the current study are available from the corresponding author on reasonable request.
