# Supplementary material for: Effect of digital based nursing intervention on knowledge of self-care behaviors and self-efficacy of adult clients with diabetes
Source: BMC Nurs. 2024 Feb 20;23:130. doi: 10.1186/s12912-024-01787-2 (PMC10877800; doi:10.1186/s12912-024-01787-2)
Supplement: Supplementary file 1 — Supplementary Material 1 [file 12912_2024_1787_MOESM1_ESM.docx]

**Diabetes Education Program**

**Objectives:**

By the end of this program, the patient will be able to:

1. Define diabetes.
2. Identify different types of diabetes.
3. Discuss the causes of diabetes.
4. Identify the symptoms of diabetes.
5. Explain the symptoms of low blood sugar.
6. Identify the symptoms of high blood sugar.
7. Explain how to monitor and conduct tests. WHICH ONES
8. State the causes of diabetic feet.
9. Explain how to care for diabetic feet.
10. Discuss how to provide psychological support to a diabetic patient with diabetic foot or foot amputation.
11. Identify diabetes medications.
12. Describe how to administer insulin injections.
13. Identify insulin injection sites.
14. Determine appropriate nutrition for diabetes.
15. List prohibited foods.
16. Explain how to prepare a complete and healthy meal for a diabetic patient. OR CLIENT?
17. Discuss suitable physical exercises for **diabetic patients**.
18. Understand how to provide proper healthcare for a **diabetic patient.**
19. Explain how to measure blood sugar levels.

2. **Introduction**

Diabetes is a lifelong companion. It is a chronic disease that requires careful and continuous management in terms of treatment, balanced nutrition for diabetic patients, and regular exercise. Sugar is your gentle enemy; if you respect it, it will respect you, but if you ignore it, your body will gradually deteriorate. Sugar is an important source of energy for the body and the primary source of energy supply for the brain. However, an increase in blood sugar levels can lead to serious health problems. Diabetes can be treated, and its consequences can be avoided or delayed by following a healthy diet, engaging in physical activity, taking prescribed medications, undergoing regular check-ups, and treating complications.

**3. Definition of Diabetes:**

Diabetes is a chronic disease that causes abnormal elevation or reduction in blood sugar levels due to a dysfunction in the secretion of insulin from the pancreas.

**4. Diabetes:**

Diabetes is characterized by the production of insufficient insulin, which is responsible for lowering blood sugar levels, due to pancreatic dysfunction.

**5. Factors that increase the risk of developing diabetes:**

- Overweight: Having excess weight increases the likelihood of developing diabetes. Excess body fat impairs the function of insulin and increases the body's resistance to it.

- Aging: The risk of developing diabetes increases, especially after the age of 45. This is partly due to changes in the body with aging and their impact on pancreatic function and response to insulin.

- Neglecting exercise and physical activity.

- Previous history of gestational diabetes in women.

- High blood pressure.

- High levels of cholesterol in the blood.

**6. General symptoms indicating that you are suffering from diabetes:**

- Frequent urination: You may experience an increased need to urinate frequently, especially during the night (nocturia).

- Excessive thirst: You may feel constantly thirsty and have an unquenchable desire for fluids.

- Unexplained weight loss: Despite normal or increased food intake, you may notice a significant and unexplained weight loss.

- Fatigue and weakness: You may feel tired and lack energy, even without engaging in strenuous activities.

- Blurred vision: You may experience blurry or distorted vision, which may affect your ability to focus clearly.

- Slow-healing wounds: Cuts, sores, or wounds may take longer to heal than usual.

- Tingling or numbness: You may experience tingling sensations or numbness in your hands or feet.

- Recurring infections: You may be prone to frequent infections, particularly in the urinary tract, skin, or gums.

- Increased hunger: Despite regular meals, you may feel hungry more often.

- Irritability: You may feel more irritable or experience mood swings.

Please note that these symptoms can vary among individuals, and it's important to consult a healthcare professional for an accurate diagnosis if you suspect you have diabetes.

**7. Symptoms of high blood sugar:**

• Feeling more thirsty than usual.

• frequent urination.

Unintentional weight loss.

• Feeling tired and weak.

• Easily irritable or other mood swings.

Blurry vision.

• Slow wound healing.

• Treatment of many infections, such as infections of the gums, skin and vagina.

**8.Signs of Low blood sugar:**

- Paleness.

• Shivering.

• Sweating.

Headache.

• Hunger or nausea.

• Rapid or irregular heartbeat.

• Exhaustion.

• Easily agitated or anxious.

• Degree of concentration.

- Dizziness or lightheadedness.

**9. How to Monitor and Perform Tests:**

**A1C Hemoglobin Test:** This test reflects the average blood sugar level over the past two or three months. Generally:

- Less than 5.7% is considered normal.
- Between 5.7% and 6.4% indicates prediabetes.
- 6.5% or higher in two separate tests indicates diabetes.
- Certain conditions can affect the accuracy of the test, such as pregnancy or the presence of abnormal hemoglobin types.

**Fasting Blood Sugar Test:**

- A blood sample is taken after fasting for at least eight hours or overnight.
- Normal level is less than 100 mg/dL (5.6 mmol/L).
- Between 100 and 125 mg/dL (5.6 to 6.9 mmol/L) indicates prediabetes.
- A level of 126 mg/dL (7.0 mmol/L) or higher in two separate tests diagnoses diabetes.

**Random Blood Sugar Test:** This test can be taken at any time without fasting. A result higher than 200 mg/dL indicates diabetes.

**10. Causes of Diabetic Foot:**

- Poor blood circulation leading to insufficient blood flow to the feet.
- High blood sugar slowing down wound healing.
- Nerve damage.
- Foot injuries.

**11. How to Care for Diabetic Foot:**

1. Prevent and care for your feet.
2. Control blood sugar levels.
3. Perform regular foot examinations, including:
   - Checking for wounds, bruises, pressure areas, redness, and nail problems. A mirror can be used for examination.
   - Sensing the foot and checking for swelling.
4. Examining between the toes.
5. Checking sensation in each foot.
6. Focusing on the six areas on the sole of each foot: the big toe, little toe, middle toe, heel, and the borders of the front part of the foot.
7. Do not attempt any treatment without consulting a doctor.
8. Foot care includes:
   - Daily washing with warm water and soap. Check the water temperature before using it.
   - Avoid soaking the feet. Dry them gently with a towel, focusing on the spaces between the toes.
   - Moisturize with moisturizing creams, avoiding applying cream between the toes.
   - Trim nails straight and avoid cutting the corners to prevent injury.
   - Immediately inform the doctor about any nail problems.
   - Avoid using sanitizers, ointments, hot compresses, or sharp tools on the feet. Keep them warm by wearing socks and avoid exposing them to heat sources.
   - Wear loose socks when sleeping.
   - Avoid direct exposure to ice and rain.
   - Avoid crossing one foot over the other for extended periods, as it hampers blood flow to the feet.
   - Avoid smoking and walking barefoot.

**9. When Choosing Shoes, Consider:**

- Avoid wearing open-toed shoes.
- Choose shoes carefully and try them on at the end of the day when your feet are larger.
- Ensure the shoe is comfortable before purchasing and check its size thoroughly.
- Avoid pointed-toe and high-heeled shoes. Select for shoes with a wide toe box.
- Do not wear the same shoes every day.
- Feel the inside of the shoe with your hand before wearing it.
- Lace the shoe moderately.
- Wear clean and dry socks, changing them daily, and avoid wearing socks with holes.

**12. Psychological Care for Diabetic Patients:**

**Diabetic patients** often experience anxiety and mood fluctuations due to fluctuating blood sugar levels. They might feel that they have an underlying issue they don't fully comprehend and struggle to manage. Family and close friends play a crucial role in encouraging the patient to lead a balanced life, avoiding making them feel sick, different, or in need of special treatment. Cooperation with the patient and working together to alleviate anxiety resulting from changing health conditions is essential. It's also important for the patient to have a sufficient understanding of their health condition and how to manage both high and low blood sugar levels. This helps in accepting the condition, reducing fear, and mitigating excessive overthinking.

The medical team is also integral in providing psychological support. They explain the nature of the disease, its treatment plan, appropriate dosages, and how to manage doses on special days or when the patient is unwell. This equips the patient to handle any health changes with readiness. It's always recommended for **diabetic patients** to connect with others who share the same condition for mutual support and exchange of experiences. The advent of social media and modern media has made it easier for patients to access interested individuals and specialists in diabetes, facilitating communication and knowledge sharing.

13. Insulin Injection Sites:

- Upper Arm Area: Place the injection behind the arm, midway between the shoulder and elbow.

- Abdominal Area: Inject insulin about 5 cm away from the navel.

- Thigh Area: Inject in the middle between the knee and hip.

**14. Steps for Insulin Injection:**

1. **Prepare the Supplies:**
   - Wash your hands thoroughly with soap and water.
   - Gather the insulin vial, syringe or pen, alcohol swab, and a sharps disposal container.
2. **Check the Insulin:**
   - Roll the insulin vial gently between your palms to mix it (if needed). Do not shake the vial vigorously.
3. **Select the Injection Site:**
   - Choose an injection site as advised by your healthcare provider. Common sites include the abdomen, thighs, and upper arms.
4. **Clean the Skin:**
   - Use an alcohol swab to clean the injection site and let it dry.
5. **Prepare the Syringe or Pen:**
   - If using a syringe, draw the correct amount of insulin into the syringe.
   - If using an insulin pen, set the dose according to your prescription.
6. **Inject the Insulin:**
   - Hold the syringe or pen like a pencil or a pen.
   - Pinch a fold of skin at the injection site (if applicable) to create a small area for the injection.
   - Insert the needle into the skin at a 90-degree angle (or as instructed by your healthcare provider).
   - Push the plunger of the syringe or press the button on the insulin pen to inject the insulin.
7. **Remove the Needle:**
   - Keep the needle in the skin for a few seconds to ensure the full dose is delivered.
   - Withdraw the needle gently and dispose of it in a sharps container.
8. **Apply Pressure and Discard:**
   - Press a clean cotton ball or gauze over the injection site for a few seconds to prevent bleeding.
   - Recap the syringe or dispose of the pen properly.
9. **Rotate Injection Sites:**
   - To avoid skin irritation and lipodystrophy (changes in the fatty tissue under the skin), rotate injection sites within the chosen area.
10. **Record the Injection:**
    - Keep a log of your insulin injections, including the dose, time, and site. This can help you and your healthcare provider track your treatment.

**15. Healthcare Strategies for Diabetic Patients:**

- Regular physical exercise helps regulate blood sugar levels.

- Best exercises for lowering blood sugar include walking, playing tennis, doing household chores, climbing stairs, and simple chair exercises like sitting and raising your arms.

- Achieve a healthy weight to counteract the insulin-resistant hormones produced by accumulated fats.

- Avoid stressors, cultivate relaxation, and ensure sufficient sleep for a calm mind.

- Practice deep breathing exercises to help with relaxation.

- Consume a diet rich in dietary fiber, including carbohydrates rich in fiber, such as whole grains, fruits, and vegetables, to stabilize glucose levels.

- Include beans, dark leafy greens, fatty fish, fruits, nuts, and whole grains in your diet for their diabetes-friendly properties.

- Consume dairy products with reduced fat and rich in calcium.

- Avoid smoking.

16. Diabetes Medications:

- Insulin Pump: A device programmed to deliver predetermined insulin amounts at specific times.

- Tablets.

- Insulin.

17. Proper Nutrition for **Diabetic Patients**:

- Consume three main meals and three healthy snacks per day.

- Distribute daily intake into three main meals (breakfast, lunch, dinner) and three healthy snacks to prevent hunger.

- A healthy snack before bedtime helps prevent nighttime hypoglycemia.

18. Creating a Balanced Meal for **Diabetic Patients**:

A balanced meal for diabetic patients should focus on providing a combination of carbohydrates, protein, and healthy fats to help stabilize blood sugar levels. Here's an example of a balanced meal:

**Grilled Chicken Salad with Quinoa:**

**1. Grilled Chicken:**

- Grilled skinless chicken breast (about 3-4 oz)

- Marinated with herbs, lemon juice, and a touch of olive oil for flavor

**2. Quinoa:**

- 1/2 cup cooked quinoa

- Quinoa is a whole grain that provides complex carbohydrates and dietary fiber, helping to regulate blood sugar.

**3. Vegetables:**

- Mixed greens (spinach, kale, arugula)

- Sliced cucumbers, bell peppers, and cherry tomatoes

- Vegetables provide vitamins, minerals, and fiber.

**4. Healthy Fats:**

- 1/4 avocado, sliced

- A source of monounsaturated fats that contribute to heart health and satiety.

**5. Dressing:**

- Homemade vinaigrette with olive oil, balsamic vinegar, Dijon mustard, and herbs.

- A balanced dressing that adds flavor and healthy fats in moderation.

**6. Nuts (Optional):**

- A small handful of almonds or walnuts

- Nuts can add crunch, healthy fats, and a source of protein.

**Beverage:**

- Water, herbal tea, or unsweetened beverage of choice

**Notes:

- This meal includes lean protein from the chicken, complex carbohydrates from the quinoa, and a variety of non-starchy vegetables.

- The healthy fats from avocado and olive oil help slow down the digestion of carbohydrates and contribute to satiety.

- Portion sizes should be adjusted based on individual calorie and carbohydrate needs.

- It's important to monitor blood sugar levels and consult with a healthcare provider or registered dietitian for personalized meal planning.

Remember that individual dietary preferences, nutritional needs, and health goals can vary. It's recommended to work with a healthcare professional or registered dietitian to create a meal plan that is tailored to your specific needs and helps you manage your diabetes effectively.

**19. Medical Care for Diabetic Patients:**

- Follow a healthy diet plan and reduce sugar.

- Exercise for 30 minutes daily.

- Take diabetes medication at the same time every day and maintain the prescribed dosage.

- Check blood sugar daily and record results.

- Examine the feet daily for wounds.

- Maintain oral hygiene and gum health.

- Quit smoking.

**References**

1. **Beyond Type 1. Forms of Diabetes.*(https://beyondtype1.org/other-forms-of-diabetes/)*Accessed 2/17/2023.**
2. **Centers for Disease Control and Prevention. Diabetes.*(https://www.cdc.gov/diabetes/index.html)*Accessed** 2/17/2023.
3. Centers for Disease Control and Prevention. National **Diabetes Statistics Report 2020.*(https://www.cdc.gov/diabetes/pdfs/data/statistics/national-diabetes-statistics-report.pdf)*Accessed 2/17/2023.**
4. **Merck Manual: Consumer Version. Diabetes Mellitus (DM).*(https://www.merckmanuals.com/home/hormonal-and-metabolic-disorders/diabetes-mellitus-dm-and-disorders-of-blood-sugar-metabolism/diabetes-mellitus-dm?query=diabetes)*Accessed 2/17/2023.**
5. **Sapra A, Bhandari P. Diabetes Mellitus.*(https://www.ncbi.nlm.nih.gov/books/NBK551501/)*2022 Jun 26. In: StatPearls [Internet]. Treasure Island, FL: StatPearls Publishing; 2022 Jan-. Accessed 2/17/2023.**
6. **U.S. National Institute of Diabetes and Digestive and Kidney Diseases. Diabetes.*(https://www.niddk.nih.gov/health-information/diabetes/)*Accessed 2/17/2023.**
7. **U.S. National Institute of Diabetes and Digestive and Kidney Diseases. What is Diabetes?*(https://www.niddk.nih.gov/health-information/diabetes/overview/what-is-diabetes)*Accessed 2/17/2023**
