# Supplementary material for: Effect of digital based nursing intervention on knowledge of self-care behaviors and self-efficacy of adult clients with diabetes
Source: BMC Nurs. 2024 Feb 20;23:130. doi: 10.1186/s12912-024-01787-2 (PMC10877800; doi:10.1186/s12912-024-01787-2)
Supplement: Supplementary file 2 — Supplementary Material 2 [file 12912_2024_1787_MOESM2_ESM.pdf]

Code:

Dear Participant,

We invite you to partake in a vital study aimed at understanding the impact of nursing interventions on self-care behaviors and self-efficacy in individuals with diabetes. Your participation will involve completing a brief questionnaire about your socio-demographic background. This information is crucial for our research and will be kept strictly confidential. We appreciate your valuable contribution to this study.

Socio-Demographic Data Questionnaire

Participant Information:

1. **Age:**
    - "Please specify your age in years."
2. **Gender:**
    - "Please indicate your gender."
        - Male
        - Female
        - Non-binary
        - Prefer not to disclose.
        - Other (please specify) ___________
3. **Duration of Diabetes:**
    - "For how many years have you been diagnosed with diabetes?"
        - Less than 1 year
        - 1-5 years
        - 6-10 years
        - 11-15 years
        - Over 15 years

Your responses are confidential and will be used solely for research purposes.
